# Comment on: Increased Theophylline Plasma Concentrations in a Patient With COVID-19

**DOI:** 10.1177/10600280241278336

**Published:** 2024-09-09

**Authors:** Evelyn Krohmer, Walter E. Haefeli

**Affiliations:** 1 Internal Medicine IX: Department of Clinical Pharmacology and Pharmacoepidemiology, Heidelberg University, Medical Faculty Heidelberg/Heidelberg University Hospital, Heidelberg, Germany

Dear Editor,

We read with great interest the recent letter to the editor by Rodríguez Fernández and co-workers,^1^ describing an 81-year-old male patient with chronic obstructive pulmonary disease who had an 81.6% increase in theophylline plasma concentrations during infection with severe acute respiratory syndrome coronavirus 2 (SARS-CoV2). As the dosing of the co-administered potential perpetrator drugs was either short (remdesivir treatment only for 3 days) or remained unchanged (allopurinol and diltiazem), the authors concluded that a drug-drug interaction was unlikely, and the decrease in clearance was caused by SARS-CoV2 infection, which can trigger a proinflammatory cytokine storm with subsequent liver injury that could decrease cytochrome P450 (CYP) expression and activity.

It has already been shown that respiratory infections,^2^ including SARS-CoV2 infection,^3^ can alter the activity of several CYP, with CYP1A2 activity being particularly frequently decreased (in 73% of cases).^3^ The extent of this inhibition can be significant, halving the metabolic ratio of the CYP1A2 marker substrate caffeine.^3^ As suspected by the authors, it is therefore possible that COVID-19 caused the change in theophylline exposure in this patient. However, as CYP1A2 activity is unchanged in about a quarter of the patients with SARS-CoV2 infection,^3^ and since this patient was also receiving metamizole, a dose-dependent inhibitor of CYP1A2,^4^ the authors’ conclusion is only plausible if the metamizole dosage was unchanged. At doses of 3 g/d, metamizole increased caffeine exposure by 79% in healthy volunteers,^4^ that is, in a similar order of magnitude as observed with theophylline in this patient. It seems quite possible that the metamizole doses varied and that higher doses of the antipyretic drug were administered during the acute phase of the illness than during the recovery phase, so that metamizole alone could have caused or at least contributed to the observed changes in theophylline exposure. It would therefore be important to report which metamizole doses were administered during and after the SARS-CoV2 infection in this patient.
